# Smoking cessation in COPD: confronting the challenge

**DOI:** 10.1007/s11739-021-02710-2

**Published:** 2021-03-25

**Authors:** Donald P. Tashkin

**Affiliations:** Department of Medicine, Division of Pulmonary and Critical Care Medicine, David Geffen School of Medicine at UCLA, 10833 Le Conte Ave, Los Angeles, CA 90095 USA

Despite clear evidence of the benefits of smoking cessation in COPD, including decreased disease progression [1, 2], symptom improvement [3] and reduced mortality [4], 30–50% of symptomatic patients with moderate to very severe COPD continue to smoke [5] and, of those who make a serious attempt to quit (counseling plus pharmacotherapy), 65–85% are still smoking at 1 year. For example, the results of randomized controlled trials of approved pharmacotherapy for smoking cessation, along with at least brief counseling, that focused on smokers with COPD, only 14 to approximately 25% succeeded in achieving sustained smoking cessation from 6 to 12 months after randomization [6–9]. Since smokers with COPD are often unable to quit completely as the best strategy for reducing the harmful effects of smoking on COPD progression, reduction in the amount of cigarettes smoked has been considered as a second-best goal with the expectation that a lower number of cigarettes smoked would still be beneficial in reducing the harm from smoking. However, data from the Lung Health Study failed to find a relationship between reduction in cigarettes smoked per day and the annual rate of decline in lung function, except for the small minority of smokers who were able to both reduce their amount of smoking to very low levels and sustain that amount of reduction [10].

In view of these discouraging findings, an American College of Chest Physicians (ACCP) panel proposed a toolkit based on a stepwise approach to treatment analogous to that recommended for asthma, e.g., in the GINA guidelines [11]. In line with smoking cessation guidelines from the U.S. Public Health Service urging physicians to consider combinations of pharmacologic aids to smoking cessation with proven effectiveness [12], this toolkit included different levels of single or combined pharmacotherapy that comprised controllers (nicotine patch, bupropion, varenicline) and relievers (rapidly acting nicotine replacement therapy) based on the pre-treatment severity of tobacco dependence and the presence and degree of withdrawal symptoms during treatment [13, 14]. The toolkit was based on the rationale that treatment needs to be tailored to the needs of individual patients with varying levels of tobacco dependence. After treatment is initiated, the intensity of pharmacotherapy can be adjusted up or down depending on the level of control of tobacco dependence. However, serious implementation of this approach requires time and dedication on the part of the practicing clinician as well as an awareness and appreciation of the importance of this approach, requirements that unfortunately may be sorely lacking in the general medical community.

An alternative, although not universally approved, approach involves the use of electronic nicotine delivery systems (ENDS), including electronic (e-) cigarettes (EC) and heated tobacco products (HTP), also referred to as heat-not burn (HnB) products. These products have potential advantages in reducing the harm from tobacco smoking as a consequence of their delivery of nicotine from a tobacco plug through a combustion-free heating system leading to markedly, albeit not totally, reduced emissions of the toxic ingredients found in tobacco smoke. However, considerable controversy continues to surround the use of these products, mainly with regard to their potential use (misuse) by nonsmoking youths and adults, arguably serving as a gateway to smoking, a controversy illustrated by recent letters to the editors of this journal characterizing these systems as a “double-edged sword” [15, 16].

To substantiate the true potential for ENDS for sustained reduction in harm from smoking, carefully designed long-term follow-up studies are required that systematically evaluate the impact of the use of ENDS to achieve smoking cessation (or marked reduction in the number of cigarettes smoked) particularly in smokers with a smoking-related disease, such as chronic obstructive pulmonary disease (COPD), in comparison with appropriate matched control smokers with COPD who continue to smoke. Appropriate outcomes of these studies include, among others, changes in lung function, respiratory symptoms, health-related quality of life, exacerbations of COPD and physical functional ability. Polosa and colleagues have recently reported the results of such a prospective study in smokers with COPD prior to and over a 5-year follow-up period after switching to ECs in comparison with a matched control group of smokers with COPD who did not use ECs [17]. Their findings indicated a significant and sustained improvement in lung function, symptoms and functional ability in the EC users compared with the reference group, likely as a result of reduction in the harmful effects of continuing smoking.

Using a similar experimental design, Polosa and colleagues have conducted the first follow-up study of the impact of using an alternative type of ENDS, namely two commercially available types of HTPs (“iQOS” and “glo”), by smokers with COPD for smoking cessation in comparison with an age- and sex-matched group of COPD patients who continued smoking (non-HTP group), the findings of which are reported in the current issue of this journal [18]. On average, the HTP group succeeded in achieving either sustained smoking cessation (~ 60% of the group) or marked reduction the number of cigarettes smoked per day, while no changes in smoking amount were noted in the control group. Thus, the switch to HTPs successfully achieved the goal of smoking cessation/reduction. While the number of patients in each study group who completed the 3-year follow-up study was small (*n* = 19), significant reductions were noted in exacerbations of COPD and improvements in symptoms, health-related quality of life and exercise capacity in the HTP group, although no changes were noted in lung function in either group. Exacerbations are a particularly important event in COPD that contributes substantially to morbidity, disease progression and mortality and are accordingly listed as one of the most important goals of COPD management [19]. Consequently, the significant reduction in exacerbation rate in this small study is a particularly notable finding. The underlying mechanism accounting for these beneficial findings is undoubtedly related to the cessation or marked reduction in regular cigarette smoking, thereby reducing exposure to the toxic proinflammatory ingredients in tobacco smoke that lead to the tissue injury and remodeling responsible for COPD and its progression, including the risk of exacerbations. Moreover, as the authors suggest, the near absence of CO in emissions from combustion-free HTPs might also have contributed to the observed improvement in exercise tolerance.

Notable weaknesses of the paper of Polosa and colleagues, as acknowledged by the authors, are the rather small size of the sample (19 in each group) and the possibility of selection bias in the recruitment process. In addition, the duration of follow-up (3 years) was relatively short, so that this factor, combined with the relatively small sample size, makes it impossible to evaluate the longer-term impact of switching from regular tobacco cigarettes to HTPs, particularly with regard to the potential impact on tobacco-related cancer development. With regard to the latter issue, it is noteworthy that while mainstream emissions of procarcinogenic compounds, including tobacco-associated polycyclic aromatic hydrocarbons and nitrosamines, are markedly reduced with HTPs, emissions of these toxicants are not completely eliminated [20–23]. Therefore, further studies of the longer-term impact of HTPs that include larger numbers of subjects are clearly warranted. The design of such studies might include outcomes not evaluated in the study of Polosa et al., including thoracic imaging (high-resolution computed tomography) with assessment of possible changes in the presence/extent of emphysema, air-trapping and airway wall thickening, as well as readily accessible biomarkers that have been associated with COPD severity and progression (blood, induced sputum, nasal brushings). In addition, such studies should include outcome measures related to patient satisfaction with HTPs since, on one hand, while patients might enjoy the similarity of the feel, smell and taste of these products to those of regular cigarettes, on the other hand, they might be annoyed by the need to clean the holder between uses of tobacco sticks, as recommended by the manufacturer, to remove residual fluid and tobacco plug debris from the heater in the device. Failure to adhere to this recommendation could lead to charring of the tobacco plug and melting of the polymer-film filter with release of highly toxic formaldehyde cyanohydrin, as recently reported by Davis et al. [24].

Regarding the role of HTPs as a means of confronting the challenge of smoking cessation, it cannot be overemphasized that resort to these products should be taken only for existing smokers who are unable to quit smoking despite having seriously attempted to quit using conventional measures, including approved pharmacotherapy and counseling, since these heat-not-burn products, while reducing the harm from tobacco smoking, have not been shown to be fully devoid of potential harm and their long-term impact on health is still uncertain.
